# Time in therapeutic range, quality of life and treatment satisfaction of patients on long-term warfarin for non-valvular atrial fibrillation: a cross-sectional study

**DOI:** 10.1186/s12955-020-01600-z

**Published:** 2020-10-20

**Authors:** Diana-Leh-Ching Ng, Natasya Marliana Bt Abdul Malik, Chee-Shee Chai, Greta-Miranda-Kim-Choo Goh, Seng-Beng Tan, Ping-Chong Bee, Gin-Gin Gan, Asri B. Said

**Affiliations:** 1 Department of Medicine, Faculty of Medicine and Health Science, University Malaysia Sarawak, Kota Samarahan, Sarawak Malaysia; 2 Department of Nursing, Faculty of Medicine and Health Science, University Malaysia Sarawak, Kota Samarahan, Sarawak Malaysia; 3 Department of Medicine, Faculty of Medicine, University of Malaya, Kuala Lumpur, Malaysia

**Keywords:** Warfarin, Time in therapeutic range, Quality of life, Treatment satisfaction, Direct oral anticoagulant

## Abstract

**Background:**

The use of warfarin in patients with non-valvular atrial fibrillation (NVAF) can be challenging. In this study, we evaluate the time in therapeutic range (TTR), health-related quality of life (HRQoL) and treatment satisfaction of patients on long-term warfarin for NVAF. The HRQoL and treatment satisfaction were compared based on the TTR.

**Methods:**

A cross-sectional study was conducted among patients on warfarin for NVAF who attended the anticoagulant clinic of a tertiary cardiology referral center in Sarawak from 1st June 2018 to 31st May 2019. Patients’ TTR was calculated by using Rosendaal technique, while their HRQoL and treatment satisfaction were assessed by using Short Form 12 Health Survey version 2 (SF12v2) and Duke Anticoagulant Satisfaction Scale (DASS), respectively.

**Results:**

A total of 300 patients were included, with mean TTR score of 47.0 ± 17.3%. The physical component summary (PCS) and mental component summary (MCS) score of SF-12v2 were 47.0 ± 9.0 and 53.5 ± 9.6, respectively. The total score for DASS was 55.2 ± 21.3, while the score for limitations (L), hassles and burdens (H&B) and positive psychological impacts (PPI) were 18.0 ± 10.0, 15.6 ± 9.1 and 21.6 ± 5.9, respectively. Seventy-three (24.3%) patients had good TTR (≥ 60%), with mean of 70.2 ± 8.7%; while 227 (75.5%) patients with poor TTR had significantly lower mean of 39.5 ± 11.9% (*p* = 0.006). There was no significant difference in the score of PCS (*p* = 0.150), MCS (*p* = 0.919) and each domain of SF-12v2 (*p* = 0.184–0.684) between good and poor TTR, except for social functioning (*p* = 0.019). The total DASS score was also not significantly different between group (*p* = 0.779). Similar non-significant difference was also reported in all the DASS sub dimensions (*p* = 0.502–0.699).

**Conclusions:**

Majority of the patients on long-term warfarin for NVAF in the current study have poor TTR. Their HRQoL and treatment satisfaction are independent of their TTR. Achieving a good TTR do not compromise the HRQoL and treatment satisfaction. Therefore, appropriate measures should be taken to optimise INR control, failing which direct oral anticoagulant therapy should be considered.

## Background

Atrial fibrillation (AF) is the commonest arrhythmia and represents substantial health care burden globally [1]. In 2010, the estimated prevalence of AF was 592 per 100,000 in men and 360 per 100,000 in woman [2]. The risk of developing AF doubles with each progressive decade and exceeds 20% by 80 years old [3]. AF is associated with a five-fold increase in thromboembolic events, particularly stroke [4]. Studies have shown that one in six embolic strokes are attributed to underlying AF [5]. Without anticoagulant, the risk of embolic stroke in patients with AF ranges between 1.9 and 18.2% [6]. The common causes of AF include hypertension, diabetes mellitus, obesity, congestive heart failure, coronary heart disease, rheumatic heart disease and valvular heart disease [7]. Non-valvular AF (NVAF) is defined as AF not due to mitral disease or metallic valves [8].

Warfarin is a vitamin K antagonist broadly used to prevent embolic stroke in patients with AF. The therapeutic efficacy of warfarin is measured in international normalised ratio (INR). The target range of INR varies depending on the indications for anticoagulation [9]. The INR range recommended by American College of Chest Physician for patients with NVAF is 2.0–3.0 [9]. The time in therapeutic range (TTR) is defined as the percentage of time the patients INR was within the target range. Patients with higher TTR value have been reported to have better outcome such as lesser stroke, major haemorrhagic events and death [10].

The use of warfarin in patients with NVAF can be challenging. First, warfarin has narrow therapeutic index, therefore patients may need to attend healthcare facilities regularly for INR monitoring and dose adjustment. Second, the daily maintenance dose of warfarin can vary among patients and in the same patients. Third, the metabolism of warfarin can be affected by certain foods, drugs and alcohol [11]. Fourth, genetic variation has been shown to influence the metabolism of warfarin [12]. In spite of these challenges, warfarin is still a popular oral anticoagulant in NVAF, mainly due to its affordability and availability.

Health-related quality of life (HRQoL) is defined as individuals’ satisfaction or happiness with an aspect of life determined by their physical, mental, emotional or social functioning [13]. It is an emerging focus among patients receiving warfarin for NVAF due to the longer life expectancy brought about by the advancement in healthcare. Treatment satisfaction is defined as individuals’ rating of important attributes of the process and outcomes of their treatment experience, which involve the interaction of expectation, preference and satisfaction [14, 15]. The HRQoL of patients on warfarin is frequently impaired due to change in their lifestyle, risk of bleeding, as well as lack of objective symptomatic relief from the medications. At the same time, their HRQoL may also be affected by their treatment satisfaction. Improving and enhancing satisfaction with the treatment regime is an important aspect in management of patients with chronic illness, such as AF [16]. Studies have shown a higher satisfaction, higher adherence, better knowledge and lower apprehension to warfarin therapy is associated with significantly better INR control [17–19].

To date, studies that assess the TTR, HRQoL and treatment satisfaction of patients taking warfarin for NVAF concurrently are still limited. Therefore, this study aims to evaluate the TTR, HRQoL and treatment satisfaction of patients taking warfarin for NVAF in a tertiary cardiology referral center located in Sarawak, a state in Malaysia. The HRQoL and treatment satisfaction of patients were also compared based on their TTR.

## Methodology

### Study design

We conducted a cross-sectional study for patients with NVAF attending the anticoagulant clinic of the Sarawak Heart Center (SHC) from 1st June 2018 to 31st May 2019. All patients included were aged 18 years and above, and have been receiving warfarin for at least past six months. Patients were excluded from the study if they had incomplete INR record, co-existing anti-platelet therapy, crossed-over from direct oral anticoagulant (DOAC), valvular heart disease, mechanical heart valves, hospital admission within one month before the interview attributed to any cause other than complications of warfarin, and unable to answer the questionnaires independently. The estimated minimum sample size for the study was 217 calculated based on the formula of cross sectional study for qualitative variables, n = Z_1−α_^2^ × p (1—p)/d2, in which reported prevalence of good TTR based on previous study (p) for Asian was 16.7%, Z_1−α_ was a constant of 1.96, and precision (d) of 0.05 [20, 21]. We obtained written informed consent from all the participants, and ethics approval from the Medical Research and Ethics Committee of the National Medical Research Registry of Malaysia (NMRR-17-1086-34402) and hospital. This study was conducted in accordance with the Declaration of Helsinki.

### Data collection and questionnaires

We identified the eligible patients from the registry of the hospital and approached them on the day of clinic visit. The demographic and clinical data were obtained via face-to-face interview, hospital case note, home-based card and electronic INR record.

The patients’ INR readings over the past six months to a year were recorded. Their TTR was calculated by using Rosendaal technique [22], which exclude the INR readings of the first six weeks after the warfarin was started. The clinical benefit of warfarin was not superior than dual anti-platelet when the TTR was less than 58% [23]. Therefore, in this study, we defined a good TTR at 60%. Hospitalisation refers to any admission due to the complications of taking warfarin, such as bleeding or thromboembolism. The comorbidities only took into account parameters in CHA_2_DS_2_-VASc, namely congestive cardiac failure, hypertension, diabetes mellitus, stroke/transient ischemic attack/thromboembolism history and vascular disease [9].

Patients were instructed to answer the Short Form 12v2 Health Survey (SF12v2) and Duke Anticoagulant Satisfaction Scale (DASS) independently to determine their HRQoL and treatment satisfaction [24, 25]. These questionnaires were available in original English version, as well as a validated Malay version [26, 27]. Patients could obtain explanation from the investigators if there was any problem with understanding the questionnaires. SF12v2 is a generic questionnaire that evaluates patients’ HRQoL in eight domains [24]. These include physical functioning (PF), role physical (RP), bodily pain (BP), general health perceptions (GH), vitality (V), social functioning (SF), role emotional (RE) and mental health (MH). The Quality Metric’s Health Outcome™ Scoring Software 5.0 is used to calculate the physical component summary (PCS) and mental component summary (MCS) from these eight domains. PCS and MCS range from 0 – 100, the higher value represent a better HRQoL. PCS tends to decrease with age, while MCS tends to increase with age [24]. DASS is a disease-specific questionnaire containing 25 items that address patients’ treatment satisfaction in three sub-dimensions, namely limitations (L), hassles and burdens (H&B) and positive psychological impacts (PPI) [25]. The L and H&B are also considered as negative impacts of oral anticoagulant. The total score of DASS range from 25 – 175, with lower value represents a better satisfaction. Both SF12v2 and DASS involve comparison between groups, and is not meant to be interpreted alone within a group as a categorical variable.

### Analysis

We presented categorical variables as percentage and continuous variables as the mean ± standard deviation (SD) or median with interquartile range. For between-groups differences, Chi-squared test or Fisher’s exact test was used for categorical variables, while independent sample t-test or the Mann–Whitney U test was used for continuous variables. ANCOVA was used to analyse HRQoL and treatment satisfaction between groups corrected for significant variables. A *p* value less than 0.05 was considered statistically significant. The analysis was performed by using Statistical Package for the Social Sciences (SPSS for Windows version 25.0, SPSS Inc., Chicago, IL, USA).

## Results

### Patient demographics and clinical characteristics

**Fig. 1:**
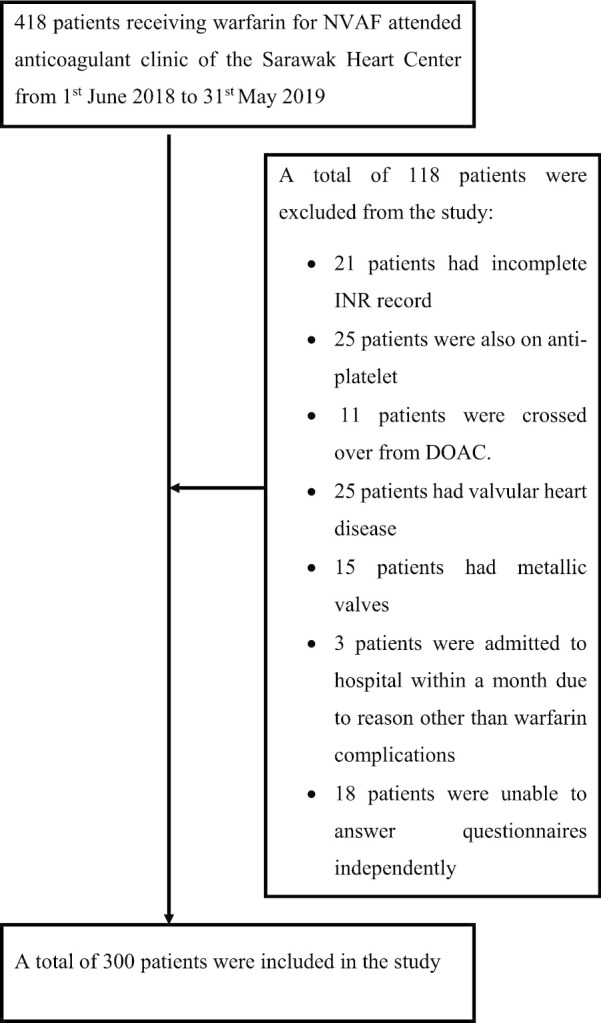
Algorithm of patients’ recruitment in the study

A total of 300 patients were included in the study (Fig. 1). Their demographic and clinical characteristics are shown in Table 1. Patients were predominantly male (53.4%), native of Sarawak (53.0%), married (73.3%), non-vegetarian/vegan (95.0%) and non-regular alcohol consumers (98.0%). Hypertension (58.7%) was the commonest comorbid, followed by congestive cardiac failure (18.0%), diabetes mellitus (17.3%) and stroke (3.7%). The mean CHA_2_DS_2_-VASc score was 2.0 ± 1.3.

**Table 1:** Demographic and clinical characteristic of patients on warfarin

Characteristics	Total patients, n = 300	Patients TTR (n, %)		*p* value
		Good 73 (24.3)	Poor 227 (75.7)	
Age (mean ± SD; 95% CI)				
Years	59.7 ± 15.6; 57.9–61.5	57.2 ± 15.0; 53.7–60.7	60.5 ± 15.7; 58.5–62.6	0.559
Gender (n, %)				
Male	172 (57.3)	39 (53.4)	133 (58.6)	0.438
Female	128 (42.7)	34 (46.6)	94 (41.4)	
Ethnicity (n, %)				
Malay	67 (22.3)	15 (20.5)	52 (22.9)	0.095
Chinese	68 (22.7)	32 (43.8)	44 (19.4)	
Native	159 (53.0)	2 (2.7)	127 (55.9)	
Others	6 (2.0)	2 (1.8)	4 (1.8)	
Partner status (n, %)				
No partner	80 (26.7)	18 (24.7)	62 (27.3)	0.420
With partner	220 (73.3)	55 (75.3)	165 (72.7)	
Education (n, %)				
None	58 (19.3)	6 (8.2)	52 (22.9)	**0.001**
Primary	101 (33.7)	19 (26.0)	82 (36.1)	
Secondary	115 (38.3)	41 (56.2)	74 (32.6)	
College/ Tertiary	26 (8.7)	7 (9.6)	19 (8.4)	
Occupation (n, %)				
Unemployed	147 (49.0)	30 (41.1)	117 (51.5)	0.269
Government dependent/ pensioner	72 (24.0)	19 (26.0)	53 (23.3)	
Private	81 (27.0)	24 (32.9)	57 (25.1)	
Diet (n, %)				
Non-vegetarian/vegan	285 (95.0)	69 (94.5)	216 (95.2)	0.829
Vegetarian/vegan	15 (5.0)	4 (5.5)	11 (4.8)	
Alcohol (n, %)				
No	294 (98.0)	71 (97.3)	223 (98.2)	0.604
Yes	6 (2.0)	2 (2.7)	4 (1.8)	
Comorbidities (n, %)				
Congestive cardiac failure*	54 (18.0)	16 (21.9)	38 (16.7)	0.317
Hypertension*	176 (58.7)	36 (49.3)	140 (61.7)	0.062
Diabetes mellitus*	52 (17.3)	11 (15.1)	41 (18.1)	0.557
Stroke/TIA//thromboembolism*	11 (3.7)	6 (8.2)	5 (2.2)	**0.017**
Vascular disease*	0	0	0	NA
Others*	67 (22.3)	12 (16.4)	55 (24.2)	0.164
CHA_2_DS_2_-VASc				
None	2.0 ± 1.3 1.8–2.1	1.8 ± 1.1 1.6–2.1	2.0 ± 1.4 1.9–2.2	0.204
Treatment duration (mean ± SD; 95% CI)				
Years	6.3 ± 6.5; 5.5–7.0	7.8 ± 8.3; 5.9–9.8	5.7 ± 5.7; 5.0–6.5	** < 0.001**
TTR (mean ± SD; 95% CI)				
%	47.0 ± 17.3; 45.0–48.9	70.2 ± 8.7; 68.2–72.2	39.5 ± 11.9; 38.0–41.1	**0.006**
Hospitalization (n, %)				
No	245 (81.7)	58 (79.5)	187 (82.4)	0.574
Yes	55 (18.3)	15 (20.5)	40 (17.6)	

*TTR* time in therapeutic range, *SD* standard deviation, *95% CI* 95% confidence interval

^*^Each of the comorbidity is over total patients

Figures in bold are statistically significant

### TTR

In this study, patients had been receiving warfarin for a mean duration of 6.3 ± 6.5 years. Their mean TTR score was 47.0 ± 17.3%. Seventy-three (24.3%) patients had good TTR, with mean score of 70.2 ± 8.7%. The mean TTR for remaining 227 (75.5%) patients were significantly lower, 39.5 ± 11.9% (*p* = 0.006). Patients with good TTR had been receiving warfarin therapy for a significantly longer duration (mean, 7.8 ± 8.3 years versus 5.7 ± 5.7 years, *p* < 0.001). The education level was significantly different between patients with good and poor TTR (*p* = 0.001). 22.9% of patients with poor TTR were uneducated, while only 8.2% of patients with good TTR did not attend school. Even though stroke/TIA/thromboembolism was significantly more common in patients with good TTR (8.2% vs. 2.2%, *p* = 0.017), the number of these patients was too small. Otherwise, there was no different in terms of patients’ gender, ethnicity, marital status, occupation, diet preference, alcohol intake and other comorbidities between patient with good and poor TTR.

### QOL

The overall PCS and MCS score were 47.0 ± 9.0 and 53.5 ± 9.6, respectively (Table 2). There was no significant difference in the score of PCS (48.3 ± 8.7 vs. 46.5 ± 9.1, *p* = 0.150), MCS (53.4 ± 8.6 vs.53.6 ± 9.7, *p* = 0.919) and each domain of SF-12v2 (*p* = 0.184–0.684) between patients with good and poor TTR, except for SF (51.5 ± 7.9 vs. 48.2 ± 11.3, *p* = 0.019). The scores for PCS (48.2 ± 1.1 vs. 46.6 ± 0.6, *p* = 0.175; 47.7 ± 1.1 versus 46.7 ± 0.6, *p* = 0.395), MCS (53.5 ± 1.1 vs. 53.5 ± 0.6, *p* = 0.998; 53.6 ± 1.1 vs. 53.5 ± 0.6, *p* = 0.951) and each domain of SF-12v2 (*p* = 0.207–0.737; *p* = 0.069–0.968) remained not significantly different between patients with good and poor TTR even after adjusted for treatment duration and education level, except for SF adjusted for treatment duration (51.9 ± 1.3 versus 48.1 ± 0.7, *p* = 0.014).

**Table 2:** Comparison of SF-12v2 score between patients with good and poor TTR

SF12v2 parameters	Total score; Mean ± SD; 95% CI	Without adjustment			Adjusted for duration*			Adjusted for education*		
		Good TTF	Poor TTF	*p* value	Good TTF	Poor TTF	*p* value	Good TTF	Poor TTF	*p* value
		Mean ± SD; 95% CI	Mean ± SD; 95% CI		Mean ± SE; 95% CI	Mean ± SE; 95% CI		Mean ± SE; 95% CI	Mean ± SE; 95% CI	
PCS	47.0 ± 9.0; 45.9–48.0	48.3 ± 8.7; 46.3–50.3	46.5 ± 9.1; 45.3–47.7	0.150	48.2 ± 1.1; 46.1–50.3	46.6 ± 0.6; 45.4–47.7	0.175	47.7 ± 1.1; 45.7–49.8	46.7 ± 0.6; 45.5–47.9	0.395
MCS	53.5 ± 9.6; 52.4–54.6	53.4 ± 8.6; 51.4–55.4	53.6 ± 9.7; 52.3–54.9	0.919	53.5 ± 1.1; 51.3–55.8	53.5 ± 0.6; 52.3–54.8	0.998	53.6 ± 1.1; 51.3–55.9	53.5 ± 0.6; 52.2–54.8	0.951
PF	47.0 ± 10.6; 45.8–48.2	48.0 ± 10.9; 45.5–50.6	46.7 ± 10.5; 45.3–48.1	0.358	47.9 ± 1.2; 45.5–50.4	46.7 ± 0.7; 45.3–48.1	0.399	47.4 ± 1.2; 45.0–49.9	46.9 ± 0.7; 45.5–48.3	0.704
RP	48.2 ± 9.6; 47.1–49.3	48.6 ± 8.8; 46.5–50.6	48.1 ± 9.9; 46.8–49.4	0.684	48.6 ± 1.1; 46.3–50.8	48.1 ± 0.6; 46.8–49.3	0.698	48.2 ± 1.1; 46.0–50.5	48.2 ± 0.6; 46.9–49.4	0.968
BP	49.6 ± 10.5; 48.5–50.8	51.1 ± 9.5; 48.8–53.3	49.2 ± 10.7; 47.8–50.6	0.184	51.0 ± 1.2; 48.6–53.4	49.2 ± 0.7; 47.8–50.6	0.207	50.5 ± 1.2; 48.0–52.9	49.4 ± 0.7; 48.0–50.7	0.446
GH	47.6 ± 9.7; 46.5–48.7	47.9 ± 9.5; 45.7–50.1	47.5 ± 9.8; 46.2–48.8	0.730	47.9 ± 1.2; 45.7–50.2	47.5 ± 0.7; 46.2–48.8	0.737	48.1 ± 1.2; 45.8–50.4	47.4 ± 0.7; 46.2–48.7	0.639
V	56.3 ± 11.3; 55.0–57.6	56.9 ± 11.5; 54.2–59.6	56.1 ± 11.3; 54.6–57.6	0.602	57.0 ± 1.3; 54.3–59.6	56.1 ± 0.8; 54.6–57.5	0.561	57.1 ± 1.3; 54.5–59.7	56.0 ± 0.8; 54.5–57.5	0.505
SF	49.0 ± 10.7; 47.8–50.2	51.5 ± 7.9; 49.7–53.4	48.2 ± 11.3; 46.7–49.6	**0.019**	51.9 ± 1.3; 49.2–54.2	48.1 ± 0.7; 46.7–49.5	**0.014**	51.0 ± 1.2; 48.5–53.4	48.3 ± 0.7; 47.0–49.7	0.069
RE	48.8 ± 10.7; 47.6–50.0	48.2 ± 10.7; 45.7–50.7	49.0 ± 10.7; 47.6–50.4	0.563	48.1 ± 1.3; 45.6–50.6	49.0 ± 0.7; 47.6–50.4	0.523	48.2 ± 1.2; 45.7–50.7	49.0 ± 0.7; 47.6–50.4	0.607
MH	53.8 ± 9.9; 52.7–55.0	53.4 ± 9.3; 51.2–55.5	54.0 ± 10.1; 52.7–55.3	0.634	53.5 ± 1.2; 51.2–55.8	54.0 ± 0.7; 52.7–55.3	0.714	53.4 ± 1.2; 51.1–55.7	54.0 ± 0.7; 52.7–55.3	0.661

*SF12v2* short form 12v2 health survey, *SD* standard deviation, *95% CI* 95% confidence interval, *PCS* physical component summary, *MCS* mental component summary, *PF* physical functioning, *RP* role–physical, *BP* bodily pain, *GH* general health perceptions, *V* vitality, *SF* social functioning, *RE* role–emotional, *MH* mental health

^*^Adjusted with ANCOVA test

Figures in bold are statistically significant

### Treatment satisfaction:

The total score for DASS was 55.2 ± 21.3, while the score for L, H&B, as well as PPI were 18.0 ± 10.0, 15.6 ± 9.1 and 21.6 ± 5.9, respectively (Table 3). The total DASS score was not significantly different between patients with good or poor TTR (54.6 ± 21.9 vs. 55.4 ± 21.2, *p* = 0.779), even after adjusted for treatment duration (55.0 ± 2.5 vs. 55.2 ± 1.4, *p* = 0.922) and education level (53.9 ± 2.5 vs. 55.6 ± 1.4, *p* = 0.563). Similar non-significant difference was also reported in all the three DASS sub dimensions (*p* = 0.502–0.699), even after adjusted for treatment duration (*p* = 0.612–0.998) and education level (*p* = 0.419–0.864).

**Table 3:** Comparison of DASS score between patients with good and poor TTR

DASS parameters	Total score, Mean ± SD; 95% CI	Without adjustment			Adjusted for duration*			Adjusted for education*		
		Good TTF	Poor TTF	*p* value	Good TTF	Poor TTF	*p* value	Good TTF	Poor TTF	*p* value
		Mean ± SD; 95% CI	Mean ± SD; 95% CI		Mean ± SE; 95% CI	Mean ± SE; 95% CI		Mean ± SE; 95% CI	Mean ± SE; 95% CI	
Total	55.2 ± 21.3; 52.6–57.6	54.6 ± 21.9; 49.5–59.7	55.4 ± 21.2; 52.6–58.1	0.779	55.0 ± 2.5; 50.0–59.9	55.2 ± 1.4; 52.5–58.0	0.922	53.9 ± 2.5; 48.9–58.9	55.6 ± 1.4; 52.8–58.4	0.563
L	18.0 ± 10.0; 16.9–19.1	18.4 ± 10.5; 15.9–20.8	17.9 ± 9.8; 16.6–19.1	0.699	18.5 ± 1.2; 16.2–20.8	17.8 ± 0.7; 16.5–19.1	0.998	17.8 ± 1.2; 15.5–20.1	18.0 ± 0.7; 16.7–19.3	0.864
H&B	15.6 ± 9.1; 14.5–16.6	14.9 ± 8.4; 13.0–16.9	15.8 ± 9.4; 14.5–17.0	0.502	15.1 ± 1.1; 13.0 -17.3	15.7 ± 0.6; 14.5–16.9	0.655	14.8 ± 1.1; 12.6–16.9	15.8 ± 0.6; 14.6–17.0	0.419
PPI	21.6 ± 5.9; 21.0–22.3	21.3 ± 6.3; 19.8–22.7	21.8 ± 5.8; 21.0–22.5	0.531	21.3 ± 0.7; 19.9–22.7	21.7 ± 0.4; 21.0–22.5	0.612	21.3 ± 0.7; 19.9–22.7	21.7 ± 0.4; 21.0–22.5	0.589

*DASS* Duke Anticoagulant Satisfaction Scale, *SD* standard deviation, *95% CI* 95% confidence interval, *L* limitations, *H&B* hassles and burdens, *PPI* positive psychological impacts

^*^Adjusted with ANCOVA test

### Hospitalisation and complications:

Fifty-five (18.3%) patients had been admitted to hospital due to complications of warfarin, all attributed to bleeding tendency. The hospitalisation rate was not significantly different between patients with good and poor TTR (20.5% vs. 17.6%, *p* = 0.574).

## Discussion

In the present study, only a quarter of patients had good TTR. Higher education level is the independent predictor of better TTR. The HRQoL of these patients was moderate, but their treatment satisfaction was good. The HRQoL and treatment satisfaction was similar irrespective of patients’ TTR. The only exception was patients with good TTR tends to have a better social functioning. Nearly one-fifth of patients had been hospitalised for bleeding tendency. Hospitalisations and bleeding tendency were independent of patients’ TTR.

Existing studies concluded patients from western countries had better TTR than their Asian counterpart. The International Study of Anticoagulant Management (INSAM) was a retrospective analysis of the real-world patients receiving warfarin for NVAF in five western countries, namely the United State, Canada, France, Italy and Spain [28]. The reported mean TTR was 57.0–68.9%, while the proportion of patients with good TTR (≥ 60%) was 47.7–75.1%. The Global Anticoagulant Registry in the Field (GARFIELD) AF reported an overall mean TTR of 55.4% and overall good TTR (≥ 65%) of 41.1% among their patients [21]. The subgroup analysis however demonstrated only 16.7% of Asian cohort had good TTR compare to 49.4% of European cohort. In another study by Chan et al., Hong Kong patients on warfarin for NVAF were reported to have a mean TTR of 38.8% and good TTR (≥ 65%) of 14.8% [29]. Oh et al. reported only 27% of Korean on warfarin for NVAF had good TTR (≥ 60%) [30]. Therefore, the lower mean TTR and the fewer good TTR among patients in this study were similar to other Asia studies. The possible explanations for this variation include Asian may have different diet and genetic, such as polymorphisms of VKOR1 and CYP2C9 genes [11, 12, 31]. The INSAM study and a meta-analysis by van Walraven et al. reported the clinical setting of warfarin given was the strongest predictor of patients TTR, being highest in randomised control trials, followed by anticoagulant clinic and community practise [28, 32]. The present study further added low education level was associated with a poorer TTR.

Regardless of the TTR, patients receiving warfarin for NVAF in the present study have markedly lower score in each domain of SF-12v2 (good TTR: 47.9–56.9; poor TTR: 46.5–56.1) when compare to Malaysia general population (SF-36, 66.7–86.0) [33], therefore significates a poorer HRQoL. To date, there is no published data that compares the HRQoL of adult patients on warfarin for NVAF based on their TTR. Most of the existing studies either compare the HRQoL of these patients versus those on DOAC, or in a longitudinal manner. Ng et al. [34], Benzimra et al. [35], Contreras et al. [36] and Alegret et al. [37] have reported no significant difference in the HRQoL of patients with NVAF receiving long-term warfarin versus DOAC in real-world scenario. On the other hand, Balci et al. reported significant improvement in every domain of SF-12v2 (all *p* < 0.001) [38], while de Caterina et al. reported significant improvement in severe mobility problem (*p* = 0.003), pain/discomfort (*p* = 0.035), and anxiety/depression (*p* < 0.001) of EuroQoL Instrument 5 levels (EQ-5D-5L) among NVAF patients after switching from warfarin to DOAC [39]. The patients in the current study had slightly higher value in each domain of the SF-12v2 except SF, but much lower mean TTR (47.0% vs. 54.9%) and proportion with good TTR (24.3% vs. 45.0%) when compared to similar subgroup of patients reported in Ng et al. [34]. Both studies were conducted in the anticoagulant clinic of a tertiary hospital, but the latter located in the Peninsular of Malaysia. The better social functioning among patients with good TTR in the current study could be due to their higher education level.

In this study, the finding of treatment satisfaction was independent of patients’ TTR was consistent with that reported in previous study. In the Outcome Registry for Better Informed Treatment of AF (ORBIT-AF) study, the treatment satisfaction of patients on warfarin for AF evaluated by using Anti-Clot Treatment scale (ACTS) was not affected by the TTR [40]. Similar finding was also observed in the Korean patients who received warfarin for NVAF when assessed by the Treatment Satisfaction Questionnaire for Medication (TSQM) [30]. A post-hoc study (ALADIN and ESPARTA) however reported significantly higher ACTS burden scale (better satisfaction) in NVAF patients with good TTR (≥ 50%) (*p* = 0.024) [19]. The cut-off point of good TTR in this study was lower than that commonly used (60–65%), therefore unable to make direct comparison with the current study.

The current study concludes that majority of the NVAF patients attending SHC had poor anticoagulant control, which may be attributed to lower education level. We recommend patients with poor TTR should be reassured that achieving a good TTR confers a better clinical outcome but does not compromise their HRQoL or satisfaction. These patients may benefit from frequent education on anticoagulant by clinicians and pharmacists during their follow-up appointments. Furthermore, the implementation of warfarin medication therapy adherence clinic (WMTAC) protocol may also help to improve patients’ INR control [41]. Switching from warfarin to DOAC should be consider in NVAF patients with poor TTR in view of the better efficacy, HRQoL and treatment satisfaction, as well as lesser side-effects. In area where the poor INR control among NVAF patients is common, DOAC should be consider as the first-line anticoagulant.

To our knowledge, this is the first study that compares the HRQoL of NVAF patients on long-term warfarin based on their TTR. Analysis of each domain of the SF12v2 and each item of the DASS were performed in order to provide a more detailed comparison. The analyses of HRQoL and treatment satisfaction were adjusted for treatment duration and education level as cofounders to minimise the result bias. This study had several limitations. Firstly, it was performed in a single centre, thus limiting the generalisability of the results. Secondly, the cross-sectional design might not be able to completely reflect the HRQoL, as HRQoL may vary over time. Thirdly, the patients HAS-BLED score and severity of bleeding was not assessed. Most of the patients were unable to remember their non-clinical significant minor bleed episode. Fourthly, patients with vascular disease taking anti-platelet were excluded. Fifthly, the TTR was calculated by using Rosendaal technique only, without comparison to the traditional method. The former assumes the changes between consecutive INR measurements are linear over time, while the latter only consider the INR value at a point of time. Finally, the treatment complications were subject to the recall bias of patients, but this was minimised by double checking available medical records.

## Conclusion

Majority of the patients on long-term warfarin for NVAF in the current study have poor TTR. Their HRQoL and treatment satisfaction are independent of their TTR. Achieving a good TTR do not compromise the HRQOL and treatment satisfaction. Therefore, appropriate measures should be taken to optimise INR control, failing which DOAC therapy should be considered. Self-management of warfarin can improve patients’ HRQoL, while adequate anticoagulant education that promote better understanding of different dosage forms, drug regime and potential side-effects can improve their treatment satisfaction.

## Data Availability

The datasets used and/or analysed during the current study are available from the corresponding author on reasonable request.

## Abbreviations

AF: Atrial fibrillation
NVAF: Non-valvular AF
INR: International normalised ratio
TTR: Time in therapeutic range
HRQoL: Health-related quality of life
SHC: Sarawak Heart Center
DOAC: Direct oral anticoagulants
TIA: Transient ischemic attack
SF12v2: Short Form 12v2 Health Survey
DASS: Duke Anticoagulant Satisfaction Scale
PF: Physical functioning
RP: Role physical
BP: Bodily pain
GH: General health perceptions
V: Vitality
SF: Social functioning
RE: Role emotional
MH: Mental health
PCS: Physical component summary
MCS: Mental component summary
L: Limitations
H&B: Hassles and burdens
PPI: Positive psychological impacts
SD: Standard deviation
INSAM: The International Study of Anticoagulant Management
GARFILED: The Global Anticoagulant Registry in the Field
EQ-5D-5L: EuroQoL Instrument 5 levels
ORBIT-AF: Outcome Registry for Better Informed Treatment of AF
ACTS: Anti-Clot Treatment scale (ACTS)
TSQM: Treatment Satisfaction Questionnaire for Medication
WMTAC: Warfarin medication therapy adherence clinic
